# The Association of GSTM1 Deletion Polymorphism with Lung Cancer Risk in Chinese Population: Evidence from an Updated Meta-analysis

**DOI:** 10.1038/srep09392

**Published:** 2015-03-23

**Authors:** Haiyan Yang, Siyu Yang, Jing Liu, Fuye Shao, Haiyu Wang, Yadong Wang

**Affiliations:** 1 Department of Epidemiology, School of Public Health, Zhengzhou University, Zhengzhou 450001, China; 2 Department of Toxicology, Henan Center for Disease Control and Prevention, Zhengzhou 450016, China

## Abstract

Previous studies have reported the association of glutathione S-transferase M1 (GSTM1) deletion polymorphism with genetic susceptibility of lung cancer in Chinese population. However, the results remained controversial. The aim of this study was to clarify the association of GSTM1 deletion polymorphism with lung cancer risk in Chinese population. Systematic searches were performed through the search engines of Medline/Pubmed, Web of Science, EMBASE, CNKI and Wanfang Medical Online. The pooled effects were calculated by STATA 10.0 software package and Review Manager 5.0.24. Overall, we observed an association of GSTM1 deletion polymorphism with increased lung cancer risk in Chinese population (odds ratio (OR) = 1.46, 95% confidence interval (95%CI): 1.32–1.66 for null genotype vs. present genotype) based on 53 studies including 7,833 cases and 10,353 controls. We also observed an increased risk of GSTM1 null genotype for lung cancer in stratified analyses by source of control, smoking status and histological type. The findings suggest that GSTM1 deletion polymorphism may contribute to lung cancer risk in Chinese population. Further, well-designed studies with larger sample sizes are required to verify the results.

The global incidence of lung cancer is 1,608,800 per year, with an annual mortality rate of 1,378,400. It was the most commonly diagnosed cancer as well as the leading cause of cancer death in males globally, and among females, it was the fourth most commonly diagnosed cancer and the second leading cause of cancer death^1^. About 85% to 90% of lung cancers are non-small cell lung cancer including squamous cell carcinoma, adenocarcinoma, large cell carcinoma and other subtypes.

Epidemiological data have shown that environmental exposures such as tobacco smoking and asbestos are the main etiological factors in lung carcinogenesis^2^,^3^. However, only a small fraction of people, who are exposed to such risk factors, will develop lung cancer. This indicates that an individual's susceptibility might play a certain role in lung carcinogenesis. Recently, increasing evidence has been accumulated to support the hypothesis that common genetic variations of drug-metabolizing enzyme genes may be of importance in determining an individual's sensitivity to develop lung cancer^4^.

Glutathione S-transferases (GSTs) are a group of phase II detoxification enzymes which detoxify a broad range of compounds, including xenobiotics, pesticides, products of oxidative stress, chemotherapeutic drugs and carcinogens such as benzo(a)pyrene and other polycyclic aromatic hydrocarbons^5^. Glutathione S-transferase mu-1 (GSTM1) is a polymorphic member of the mu class gene family of the GSTs. GSTM1 deletion polymorphism has been shown to result in the elimination of the activity of GSTM1 enzymes and modulate lung cancer risk^6^. To date, results from epidemiological studies on the association between GSTM1 deletion polymorphism and lung cancer risk in Chinese population have been mixed^7^,^8^,^9^,^10^,^11^,^12^,^13^,^14^,^15^,^16^,^17^,^18^,^19^,^20^,^21^,^22^,^23^,^24^,^25^,^26^,^27^,^28^,^29^,^30^,^31^,^32^,^33^,^34^,^35^,^36^,^37^,^38^,^39^,^40^,^41^,^42^,^43^,^44^,^45^,^46^,^47^,^48^,^49^,^50^,^51^,^52^,^53^,^54^,^55^,^56^,^57^,^58^,^59^. Recently, two meta-analyses have reported the association of GSTM1 deletion polymorphism with increased lung cancer risk in Chinese population^60^,^61^. Unfortunately, some overlapping articles were not excluded and several published papers were missing in their papers. In order to obtain a more precise estimation of this relationship, a meta-analysis including a total of 53 studies was conducted, which may provide more comprehensive evidence for the association of GSTM1 deletion polymorphism with lung cancer risk in Chinese population.

## Methods

### Literature and methods

Systematic searches were performed in Medline/Pubmed, Web of Science, EMBASE, Chinese National Knowledge Infrastructure (CNKI) and Wanfang Medical Online, with the following terms utilized: “lung cancer” or “lung tumor” or “lung carcinoma” or “non-small cell lung cancer” or “small cell lung cancer” and “polymorphism” and “GSTM1” and “Chinese” or “China”. All publications were updated to July 15, 2014. Additional relevant references quoted in the searched articles were also selected.

Criteria of literature inclusion were (a) the subjects of literature must be Chinese; (b) the papers should evaluate the association of GSTM1 deletion polymorphism with lung cancer risk; (c) case-control studies or cohort studies; (d) studies should have sufficient data for estimating odds ratio (OR) with 95% confidence intervals (CI). The exclusion criteria were (a) studies without the number of case and control or other essential information and (b) reviews and repeated or overlapping studies. For repeated studies or overlapping studies, the publication with more information was selected when more than one article was identified for the same study population.

**Figure 1:**
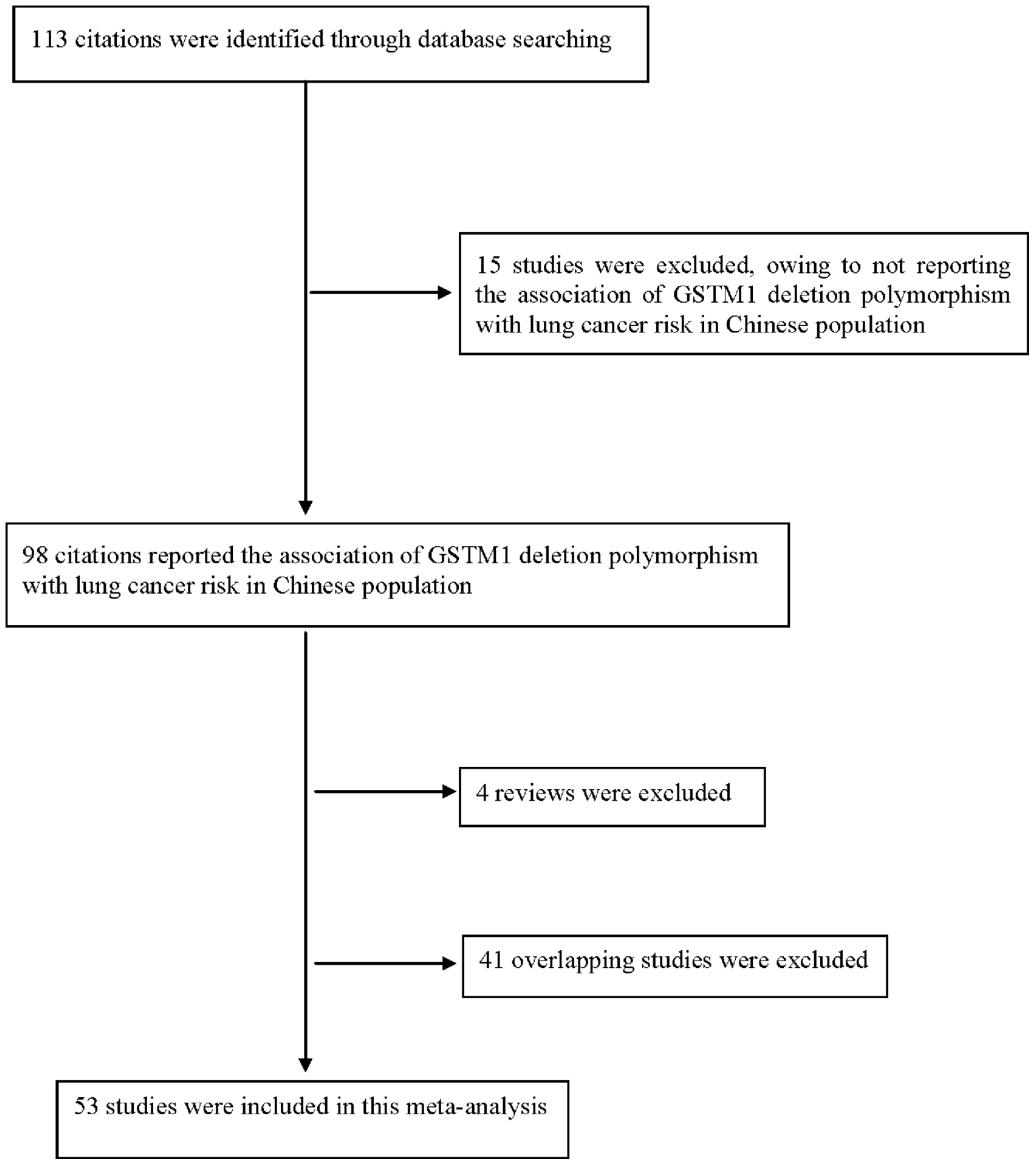
Flow diagram of selection process.

In total, ninety eight published articles were identified with the association of GSTM1 deletion polymorphism with lung cancer risk in Chinese population. We reviewed all papers according to the criteria listed, above; forty one overlapping studies and four reviews were excluded. At last, fifty three original articles that focused on the association between GSTM1 deletion polymorphism and lung cancer risk in Chinese population were determined to be eligible to enter our study (Fig. 1 Flow diagram).

### Data extraction

Data were carefully extracted from all selected articles by two of the authors, independently. The following information was subtracted from selected studies: author's name, publishing date, area, source of control, number of case and control, and number of null and present genotypes. Data coming from similar stratum were combined to make full use of them if the study provided stratum information. Characteristics of selected studies were summarized in Table 1.

### Quantitative data synthesis

The strength of the association between GSTM1 deletion polymorphism and lung cancer risk was measured by OR with 95%CI. The Cochrane *Q* statistics test was used to assess heterogeneity. The combined OR was estimated using both a fixed-effects model and a random-effects model^62^. The fixed-effects model was used when there was lack of heterogeneity. Otherwise, the random-effects model was used. The potential publication bias was firstly evaluated by visual inspection of the funnel plot. An asymmetric plot indicates that a possible publication bias exists. The funnel plot asymmetry was evaluated by the methods of Egger's test and Begg's test^63^,^64^.

Statistical analysis was done using Review Manager (Version 5.0.24, the Cochrane Collaboration) and STATA10.0 software package (Stata Corporation, College Station, Texas). All the tests were two-sided, a *P* value of less than 0.05 for any test or model was considered to be statistically significant.

## Results

### Meta-analysis databases

A database was built in the light of the extracted information from selected articles. Some essential information was listed in Table 1, which indicated the first author's name, year of publication, area, source of control, the number of case and control, and stratified factors. There were a total of 53 studies with 7,833 cases and 10,353 controls concerning the GSTM1 deletion polymorphism related to lung cancer risk. The frequency of GSTM1 null genotype was 57.7% and 50.1% in case and control, respectively.

### Test of heterogeneity

The heterogeneity of GSTM1 null genotype vs. present genotype was analyzed for 53 selected studies. The results showed that GSTM1 null genotype vs. present genotype for squamous cell carcinoma, hospitalized patients-based control, smokers and nonsmokers had no heterogeneity with a *P* value ≥0.05. Therefore, a fixed-effects model was used to calculate the summary ORs for them. A random-effects model was used to calculate the summary ORs for the rest.

### Quantitative data synthesis

**Figure 2:**
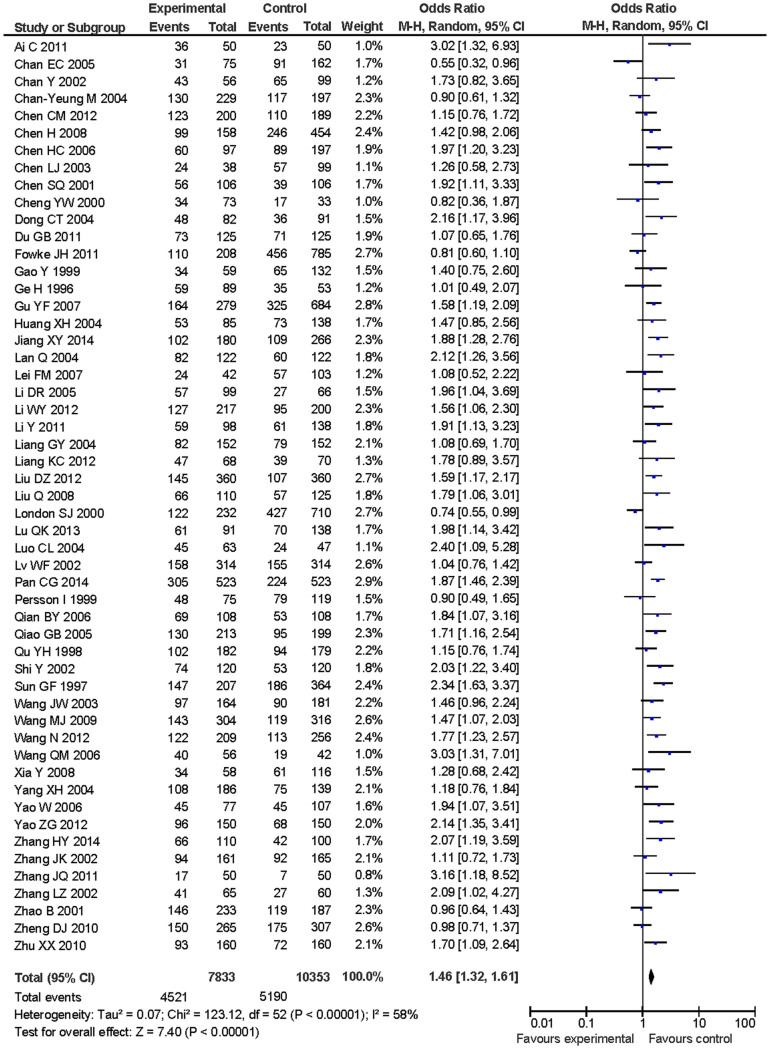
Forest plot of odds ratio for GSTM1 deletion polymorphism associated with lung cancer risk in Chinese population.

Table 2 listed the summary ORs of GSTM1 deletion polymorphism related to lung cancer risk in Chinese population on the basis of 7,833 cases and 10,353 controls. We observed an association of GSTM1 deletion polymorphism with increased lung cancer risk in the total population (OR = 1.46, 95%CI: 1.32–1.61 for null vs. present) (Fig. 2). In subgroup analysis for source of control, we observed an increased risk of lung cancer with GSTM1 null genotype in healthy subjects-based control (OR = 1.48, 95%CI: 1.32–1.66) and hospitalized patients-based control (OR = 1.40, 95%CI: 1.22–1.60), respectively. We also observed an increased risk of GSTM1 null genotype for lung cancer stratified by smoking status (OR = 1.60, 95%CI: 1.41–1.81 for smokers and OR = 1.79, 95%CI: 1.54–2.08 for nonsmokers, respectively). We observed an association between GSTM1 null genotype and increased lung cancer risk in stratified analysis by histological type (OR = 1.50, 95%CI: 1.31–1.72 for squamous cell carcinoma and OR = 1.36, 95%CI: 1.08–1.70 for adenocarcinoma, respectively) (Table 2).

### Bias diagnosis

**Figure 3:**
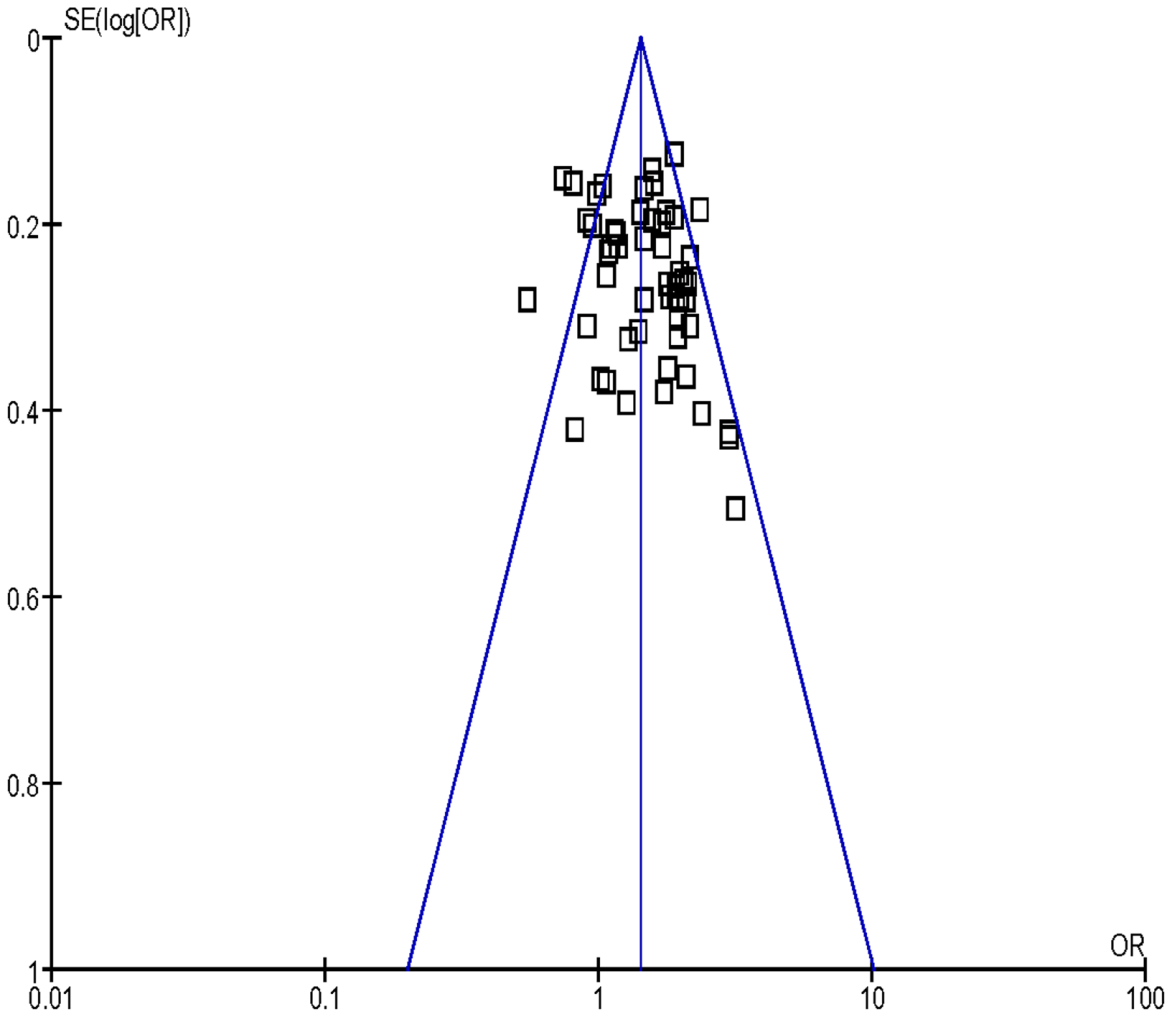
Funnel plot analysis to detect publication bias for GSTM1 deletion polymorphism associated with lung cancer risk in Chinese population.

Funnel plot was used to assess the publication bias, the shape of funnel plot seemed to be approximately symmetrical (Fig. 3). Results from Egger's test and Begg's test indicated that no obvious publication bias existed in this meta-analysis (Table 2).

### Sensitivity analysis

**Figure 4:**
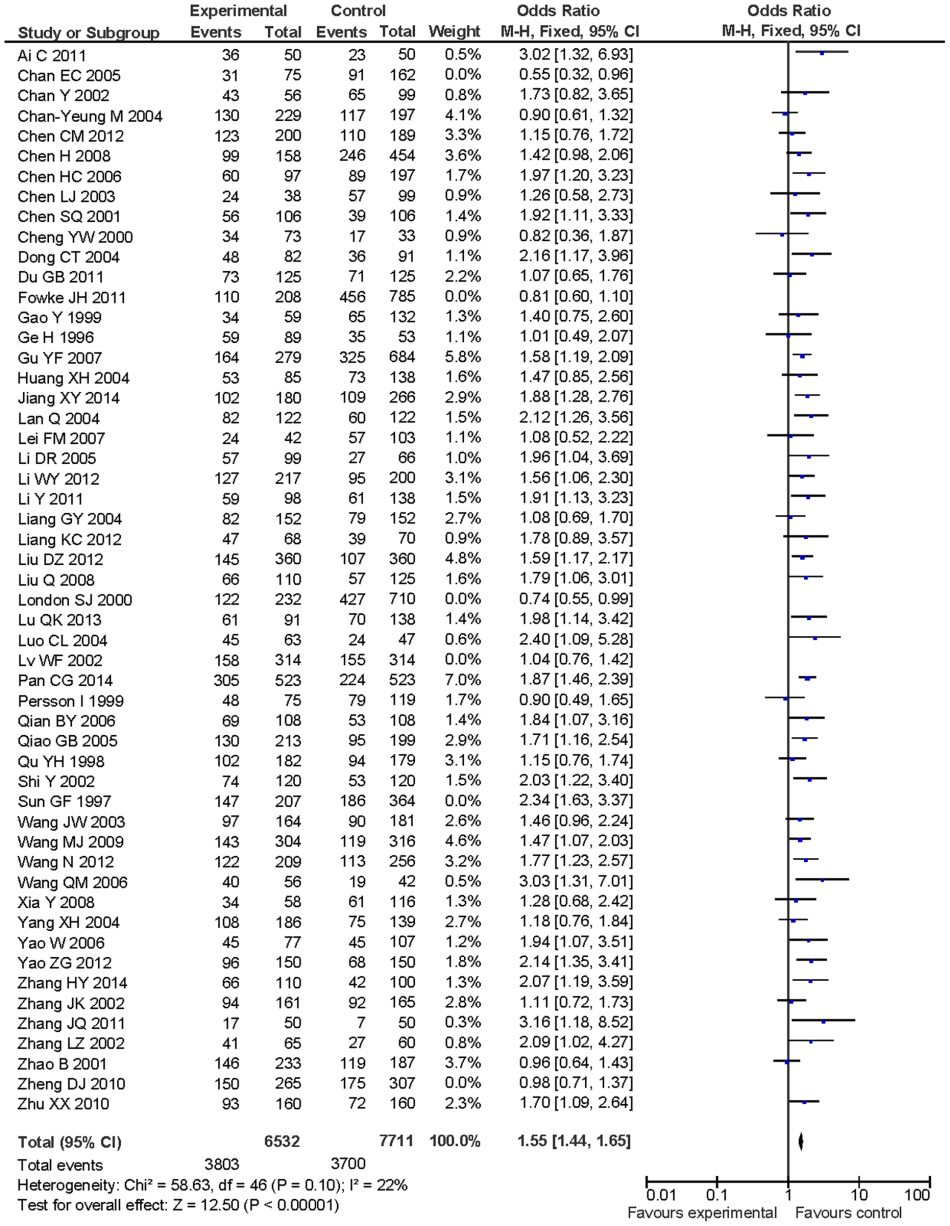
Sensitivity analysis for GSTM1 null genotype vs. present genotype in Chinese population.

The sensitivity analysis was performed to determine the influence of the individual dataset on the summary ORs by consecutively excluding individual studies. The overall effects were not changed significantly when the study was homogenous for GSTM1 null genotype vs. present genotype among total population by removing some eligible studies, indicating that our results were statistically robust (Fig. 4).

## Discussion

GSTM1 gene is located on the short arm of chromosome 1 (1p13.3)^65^. It is 5,950 bp long consisting of seven introns and eight exons, which encodes a cytosolic protein of 218 amino acid residues with a molecular weight of 21/25 kDa. GSTM1 gene has a null variant allele, which results in an absence of enzyme activity. Individuals who carry homozygous deletions in this gene are thought to be increased risks for malignancies because of their reduced capacity to detoxify potential carcinogens^66^,^67^. In addition, GSTM1 null/present polymorphisms could predict the treatment response of the platinum-based chemotherapy in NSCLC patients, especially in East-Asian patients^68^. Some meta-analyses explored the association of GSTM1 null genotype with the development of several kinds of cancers in Chinese population^69^,^70^,^71^,^72^. In this paper, we performed a systematic literature review to comprehensively evaluate the association of GSTM1 deletion polymorphism with lung cancer risk in Chinese population. We also evaluated the possible effect modifications by source of control, smoking status and histological subtype. The frequency of GSTM1 null genotype was 57.7% (range: 34%~76.7%) and 50.1% (range: 14%~66.4%) in case and control, respectively. The highest frequency of GSTM1 null genotype (66.4%) in control was found in Beijing^38^ and the lowest frequency of GSTM1 null genotype (14%) in control was found in Yunnan^55^. In summary, we observed an increased lung cancer risk in subjects with GSTM1 null genotype. Two previous meta-analyses have reported the association of GSTM1 deletion polymorphism with increased lung cancer risk in Chinese population^60^,^61^. However, there are some key limitations in their studies. For example, three overlapping studies^73^,^74^,^75^ were not properly excluded from Shi et al' study and seven papers published before 2006^13^,^16^,^41^,^42^,^43^,^54^,^56^ were missing. For Liu et al' paper, eighteen overlapping papers^74^,^76^,^77^,^78^,^79^,^80^,^81^,^82^,^83^,^84^,^85^,^86^,^87^,^88^,^89^,^90^,^91^,^92^ were not properly excluded. Therefore, the findings from these two meta-analyses should be clarified urgently by using the updated data. The present meta-analysis of 53 published studies including 7,833 cases and 10,353 controls might present a precise estimation of the association of GSTM1 deletion polymorphism with lung cancer risk in Chinese population, owing to including the updated data.

Considering that cigarette smoking is an evident risk factor for lung cancer, and that GSTM1 is involved in the metabolism of various carcinogens present in cigarette smoking, a subgroup analysis regarding smoking status was conducted. After being stratified by smoking status, the GSTM1 null genotype was associated with an increased risk of lung cancer in both smokers and nonsmokers.

Lung cancer consists of at least three major histological subtypes: squamous cell carcinoma, adenocarcinoma and small cell carcinoma. It is well-known that the development of squamous cell carcinoma and small cell carcinoma is strongly correlated with cigarette smoking, whereas that of adenocarcinoma is less correlated compared with those two subtypes, which indicates that carcinogenic processes are different among the different subtypes of lung cancer^93^. Therefore, a stratified analysis was conducted by histological subtype. We observed significant associations of GSTM1 deletion polymorphism with the increased risk of both squamous cell carcinoma and adenocarcinoma. Further stratified analyses were not done in additional histological subtypes, since the sample size for them was relatively small.

This meta-analysis should be interpreted within the context of its potential limitations. First, the combined ORs were based on individual unadjusted estimates, while a more precise analysis depending on adjusted factors should be performed if detailed individual data were available. Secondly, only published papers were enrolled in this study, which may cause publication bias. To address this issue, Egger's test and Begg's test were conducted at the same time. Our findings demonstrated that the likelihood of key publication bias might not be present in this meta-analysis. Thirdly, each study had different eligibility criteria for subjects and different source of controls, which should be taken into account while expounding the combined effects. When subgroup analysis was performed by source of control, we observed an association between GSTM1 deletion polymorphism and increased lung cancer risk in both healthy subjects-based control and hospitalized patients-based control.

In conclusion, this comprehensive review demonstrates that GSTM1 null genotype might be a risk factor for lung cancer in the Chinese population. Large scale studies with the pooling of individual study data should be taken into consideration in the future studies to verify the results from this present meta-analysis.

## Author Contributions

Conceived and designed the experiments: W.Y. and Y.H.; Performed the experiments: Y.S., S.F. and W.H.; Analyzed the data: Y.H. and L.J.; Contributed reagents/material/analysis tools: Y.S., S.F. and W.H.; Wrote the main manuscript text: W.Y. and Y.H.; Reference collection and data management: L.J. and Y.S.; Statistical analyses and paper writing: Y.H. and W.Y.; Study design: W.Y. and Y.H.; Prepared figures 1–4: W.H.; All authors reviewed the manuscript.

## Figures and Tables

**Table 1:** Studies on the association between GSTM1 deletion polymorphism and lung cancer risk in Chinese population included in this study

Author	Year	Area	Source of control	Number of case	Number of control	Stratified factors
Ai C^7^	2011	Sichuan	Healthy subjects	50	50	
Chan EC^8^	2005	Taiwan	Healthy subjects	75	162	Sex
Chan Y^40^	2002	Yunnan	Healthy subjects	56	99	
Chan-Yeung M^9^	2004	Hong Kong	Healthy subjects	229	197	Histological type
Chen CM^10^	2012	Zhejiang	Healthy subjects	200	189	Smoking
Chen H^11^	2008	Anhui	Healthy subjects	158	454	Smoking
Chen HC^12^	2006	Hunan	Healthy subjects	97	197	
Chen LJ^13^	2003	Anhui	Healthy subjects	38	99	Smoking
Chen SQ^14^	2001	Hubei	Healthy subjects	106	106	Smoking and age
Cheng YW^15^	2000	Taiwan	Hospitalized patients	73	33	
Dong CT^16^	2004	Sichuan	Hospitalized patients	82	91	
Du GB^17^	2011	Sichuan	Hospitalized patients	125	125	Histological type and smoking
Fowke JH^18^	2011	Shanghai	Healthy subjects	208	785	
Gao Y^19^	1999	Guangdong	Hospitalized patients and healthy subjects	59	132	Histological type and smoking
Ge H^20^	1996	Hongkong	Hospitalized patients and healthy subjects	89	53	
Gu YF^21^	2007	Beijing	Hospitalized patients and healthy subjects	279	684	Histological type and smoking
Huang XH^22^	2004	Guangdong	Hospitalized patients and healthy subjects	85	138	Histological type and smoking
Jiang XY^23^	2014	Inner Mongolia	Healthy subjects	180	266	
Lan Q^24^	2004	Yunnan	Healthy subjects	122	122	
Lei FM^25^	2007	Sichuan	Healthy subjects	42	103	Smoking and drinking
Li DR^26^	2005	Sichuan	hospitalized patients	99	66	Smoking
Li WY^27^	2012	Beijing	Healthy subjects	217	200	Smoking
Li Y^28^	2006	Henan	Healthy subjects	98	138	Histological type and smoking
Liang GY^29^	2004	Jiangsu	Hospitalized patients	152	152	Histological type
Liang KC^30^	2012	Guangxi	Hospitalized patients	68	70	
Liu DZ^31^	2012	Heilongjiang	Healthy subjects	360	360	Histological type and smoking
Liu Q^32^	2008	Shandong	Healthy subjects	110	125	
London SJ^33^	2000	Shanghai	Healthy subjects	232	710	
Lu QK^34^	2013	Guangdong	Healthy subjects	91	138	Histological type and smoking
Luo CL^35^	2004	Guangdong	Healthy subjects	63	47	
Lv W^36^	2002	Beijing	Healthy subjects	314	314	Histological type and smoking
Pan CG^37^	2014	Jiangxi	Healthy subjects	523	523	Histological type, smoking and sex
Persson I^38^	1999	Beijing	Healthy subjects	75	119	
Qian BY^39^	2006	Tianjin	Healthy subjects	108	108	Smoking
Qiao GB^41^	2005	Guangdong	Hospitalized patients and healthy subjects	213	199	Smoking
Qu YH^42^	1998	Shanghai and Heilongjiang	Healthy subjects	182	179	
Shi Y^43^	2002	Hubei	Hospitalized patients	120	120	
Sun GF^44^	1997	Liaoning	Healthy subjects	207	364	Smoking, age and sex
Wang JW^45^	2003	Beijing	Healthy subjects	164	181	Smoking
Wang MJ^46^	2009	Inner Mongolia	Healthy subjects	304	316	
Wang N^47^	2012	Henan	Healthy subjects	209	256	
Wang QM^48^	2006	Hubei	Healthy subjects	56	42	Smoking
Xia Y^49^	2008	Gansu	Hospitalized patients	58	116	Smoking
Yang XH^50^	2004	Liaoning	Healthy subjects	186	139	
Yao W^51^	2006	Henan	Healthy subjects	77	107	Histological type
Yao ZG^52^	2012	Beijing	Healthy subjects	150	150	Smoking
Zhang HY^53^	2014	Yunnan	Healthy subjects	110	100	
Zhang JK^54^	2002	Guangdong	Healthy subjects	161	165	Histological type and smoking
Zhang JQ^55^	2011	Yunnan	Healthy subjects	50	50	Smoking
Zhang LZ^56^	2002	Jiangsu	Healthy subjects	65	60	Histological type and smoking
Zhao B^57^	2001	Singapore	Hospitalized patients	233	187	
Zheng DJ^58^	2010	Tianjin	Healthy subjects	265	307	Histological type
Zhu XX^59^	2010	Hunan	Healthy subjects	160	160	

**Table 2:** Summery odds ratios on the relation of the GSTM1 deletion polymorphism to lung cancer risk in Chinese population

Null vs. Present	Case/Control	Heterogeneity test		Summery OR (95% CI)	Hypothesis test			Begg's test		Egger's test	
		*Q*	*P*		*Z*	*P*	*df*	*Z*	*P*	*t*	*P*
All studies	7833/10353	123.12	<0.00001	1.46 (1.32–1.61)	7.40	<0.00001	52	1.53	0.127	1.79	0.079
Stratification by source of control											
Healthy subjects	6459/8420	108.7	<0.00001	1.48 (1.32–1.66)	6.56	<0.00001	41	1.82	0.069	1.94	0.059
Hospitalized patients	1735/1933	14.88	0.31	1.40 (1.22–1.60)	4.77	<0.00001	13	0.07	0.945	0.67	0.517
Stratification by smoking status											
Yes	2284/2078	22.38	0.44	1.60 (1.41–1.81)	7.48	<0.00001	22	0.05	0.958	0.50	0.620
No	1468/2260	26.58	0.11	1.79 (1.54–2.08)	7.58	<0.00001	19	1.27	0.205	1.39	0.180
Stratification by histological Type											
Squamous cell carcinoma	1218/3375	15.96	0.25	1.50 (1.31–1.72)	5.89	<0.00001	13	0.00	1.000	0.40	0.694
Adenocarcinoma	1150/3368	28.44	0.008	1.36 (1.08–1.70)	2.66	0.008	13	0.99	0.324	0.79	0.443
